# Associations of ***PART1*** and ***DEFB1*** polymorphisms with Dental Caries in twelve-year-old children in Southern China: a cross-sectional study

**DOI:** 10.1186/s12887-022-03678-4

**Published:** 2023-01-04

**Authors:** Fei Ma, Haoyu He, Shaoyong Chen, Xueting Yu, Qiulin Liu, Xiaojuan Zeng

**Affiliations:** 1 Department of Dental Public Health, College of Stomatology, Hospital of Stomatology, Guangxi Medical University, No. 10 Shuangyong Road, 530021 Nanning, Guangxi China; 2 Guangxi Health Commission Key Laboratory of Prevention and Treatment for Oral Infectious Diseases, Nanning, Guangxi China

**Keywords:** Dental caries, Gene polymorphisms, Caries prevalence

## Abstract

**Objective:**

The aim of this study was to assess associations of *PART1* rs27565 and *DEFB1* rs11362 polymorphisms with the prevalence of dental caries in twelve-year-old children in Nandan County, Guangxi, China.

**Methods:**

A total of 1,061 children were included in this cross-sectional study and divided into two groups based on the Decayed, Missing and Filled teeth (DMFT) index: caries-free children (DMFT score = 0) and children with caries (DMFT score ≥ 1). Demographic characteristics, oral hygiene behaviour and dietary habits were collected through household records and questionnaires. Genomic DNA was extracted from buccal cells, and *PART1* rs27565 and *DEFB1* rs11362 polymorphisms were genotyped using a custom-designed 48-Plex single nucleotide polymorphism-scan kit.

**Results:**

Carriers of the *PART1* rs27565 C allele (odds ratio [OR] = 1.338, 95% confidence interval (CI) = 1.015–1.764, *P* value = 0.039) and carriers of the *DEFB1* rs11362 T allele (OR = 1.364, 95% CI = 1.056–1.762, *P* value = 0.017) had a higher risk of caries. Carriers of the *PART1* rs27565 TC or CC genotype who ate sugary food more than once a week had a 1.6-fold higher risk of caries than TT carriers who ate sugary food at most once a week (OR = 1.579, 95% CI = 1.032–2.414, *P* value = 0.035). Carriers of the *DEFB1* rs11362 CT or TT genotype who ate sugary food more than once a week had a 2.1-fold higher risk of caries than CC carriers who ate sugary food at most once a week (OR = 2.057, 95% CI = 1.438–2.940, *P* value < 0.001).

**Conclusion:**

*PART1* rs27565 and *DEFB1* rs11362 polymorphisms were associated with caries in 12-year-old children in Nandan County, Guangxi, China. Carriers of the *PART1* rs27565 TC or CC genotype and the *DEFB1* rs11362 CT or TT genotype who ate sugary food more than once a week had a high probability of having caries.

## Introduction

Dental caries is a major public health problem worldwide. Untreated caries in permanent teeth was the most prevalent health condition, affecting 35% of the global population; untreated caries in deciduous teeth was the tenth most prevalent health condition, affecting 9.0% of the global paediatric population [1]. Caries may cause toothache, masticatory dysfunction and psychological disorders and impact people’s quality of life [1–4]. Overall, the global burden of untreated caries in deciduous and permanent teeth has remained constant over the past 30 years, despite various measures implemented to prevent and control caries [1]. Thus, studying the factors affecting the prevalence of caries is very important.

Dental caries is likely influenced by multiple factors, such as the oral microbiota, sugars, tooth structure, salivary and fluoride exposure, socioeconomic status, oral hygiene habits and genetic factors [2, 5]. Among these factors, the oral microbiota plays a key role in the aetiology of caries. The ecological plaque hypotheses, which are now generally accepted as the most plausible explanations for the microbial aetiology of caries, consider that caries is a consequence of an unfavourable shift in the balance of the resident microbiota driven by changes in the dental environment [2, 6]. Caries will not occur in the absence of a cariogenic dental biofilm composed of complex microbial communities [2].

Studies have shown that the oral microenvironment and the development of dental biofilms are affected not only by diet and oral hygiene habits but also by natural immune factors. For example, individuals with very low levels of saliva antibodies or proteins are more sensitive to plaque accumulation and subsequent caries formation [7, 8]. The structures, functions and levels of these antibodies or proteins might be influenced by genetic variants [9, 10].

*PART1* and *DEFB1* are two of the immune-related genes [11, 12]. This study focused on *PART1* rs27565 and *DEFB1* rs11362 polymorphisms due to their previous potential association with dental caries. *PART1* rs27565 is located on chromosome 5q13.3, which was reported to be associated with a lower caries susceptibility by Vieira using a genome-wide linkage scan [13]. mRNA expression of *PART1* has been detected in saliva, salivary glands, and the prostate [12]. The *PART1* rs27565 T allele was associated with low caries susceptibility in Filipino families [12]. The study by Kelly also reported a higher frequency of the C allele in the caries group [14], while another study showed that the association between the *PART1* rs27565 polymorphism and the risk of caries was not significant after the Bonferroni adjustment of the *P* value [15]. *DEFB1*, consisting of two exons and one intron, encodes β-defensin 1, which is synthesized as a 64–68 amino acid prepropeptide and is expressed in the major salivary glands, tongue, gingiva, and buccal mucosa [16, 17]. β-Defensin 1 has broad-spectrum antibacterial (against gram-positive and gram-negative bacteria), antiviral and antifungal activities, protecting individuals from pathogens [18]. The rs11362 is located in the 5′ untranslated region (5`-UTR) of the reference sequence of *DEFB1* (NM_005218) at the − 20 (c.-20G > A) position [19]. A relationship between the *DEFB1* rs11362 polymorphism and caries was observed in some populations. Carriers of the *DEFB1* rs11362 T allele have a higher risk of caries than carriers of the C allele, as documented in studies of children from Ribeirão Preto in Brazil [20] and Gansu Province in China [19], as well as in adults from Turkey [21] and North America [22]. In contrast, studies examining Italian adults and Latvia children with cleft lip suggested that CC genotype carriers have a higher risk of caries [23, 24]. In addition, some studies did not report any association between the rs11362 polymorphism and caries [25, 26].

Although the relationship between gene polymorphisms and caries has been studied for decades and genetic factors play a well-established role in an individual’s risk of caries, the results of studies are inconsistent, potentially due to differences in the study populations [27]. Therefore, research on the relationship between gene polymorphisms and caries prevalence in diverse populations with various geographic origins and different ages will advance our understanding of the role of genetics in caries development and/or progression [27]. Nandan County, located in northwestern Guangxi Province in southern China, is an area containing ethnic minority groups and thus has a population that exhibits large differences in genetic backgrounds and living habits compared with those of other populations. Nandan is a remote mountainous area, and people live in relative isolation because of the poor construction of infrastructure facilities and the traffic inconvenience. Although the inhabitants belong to different ethnic subgroups, they share the same ancestry and have relatively consistent genetic backgrounds. Studies assessing the correlation between gene polymorphisms and dental caries in this area are still scarce. Therefore, the purpose of this study was to explore the relationships of *PART1* rs27565 and *DEFB1* rs11362 polymorphisms with caries prevalence in 12-year-old children in Nandan County.

## Subjects and methods

### Subjects

Twelve-year-old children in Nandan County, Guangxi (water and milk are not fluoridated) were selected as the study population. The expected prevalence of caries in 12-year-old children residing in Guangxi was 43.8% [28]. The sample size was calculated by using Quanto program (Version 1.2.3, https://bio.tools/QUANTO) based on the association between the prevalence of caries and the frequency of the risk allele in the population. The frequencies of the *PART1* rs27565 C allele and *DEFB1* rs11362 T allele, which are potentially associated with high caries risk, are 0.48 and 0.40 in Asian populations, respectively (HaploReg v4.1, https://broadinstitute.org). It was calculated that at least 974 participants would be required with α = 0.05, 1-β = 0.8 and expected odds ratio = 1.5. The expected odds ratio was determined by the results from previous studies [13, 20]. This calculated sample size also met the statistical requirements of binary logistic regression analysis in this study. Then, fifteen of 55 primary schools in Nandan County were selected using a random number generator software program (Microsoft Office Excel 2010, USA). Twelve-year-old children from the selected schools who were free of chronic diseases and unrelated were recruited. Finally, 1,061 children were included after excluding subjects with incomplete data.

This study was approved by the Institutional Research Ethics Committee of Guangxi Medical University. All the children assented to participate in this survey on-site, and guardians provided written informed consent. All methods were conducted in accordance with relevant guidelines and regulations.

### Data collection

Clinical examinations were performed by three dentists to collect data on caries experience. The dentists were trained by an experienced dental epidemiologist. The inter- and intra-examiner consistency test was conducted following the protocol suggested by the World Health Organization (WHO) [29]. Thirty participants were assessed and the kappa test was used to observe the consistency of measurements with each examiner.

The experience of dental caries in permanent teeth was recorded using the Decayed, Missing and Filled teeth (DMFT) index according to the standard recommended by the WHO in 2013 [29]. Children were divided into two groups based on the DMFT index: caries-free children (DMFT score = 0) and children with caries (DMFT score ≥ 1).

Demographic characteristics, such as sex, ethnicity (defined by both parents being of the same ethnic group), age and parental education level (the highest education level attained by the parents) were obtained from household records. Data on oral hygiene behaviour and dietary habits were collected using a structured questionnaire designed with reference to the Fourth Chinese National Oral Health Survey [30]. Dietary habits were assessed, specifically whether the children regularly ate at school and the frequency of sugary food intake. Sugary food was defined as sweet foods, sweetened milk/yoghurt/tea/coffee and other sweet drinks [30]. The frequency of sweet food intake was recorded in six categories (0 = seldom, 1 = one to three times per month, 2 = once per week, 3 = two to six times per week, 4 = once per day, and 5 = more than twice per day) [30] and then divided into two groups: at most once a week (with scores of 0, 1 and 2) and more than once a week (with scores of 3, 4 and 5). Structured questionnaires were completed by children and reviewed by research assistants.

### DNA isolation and genotyping

Subjects were asked to gargle and do not eat or drink within 30 min before sampling to ensure that the sample was not contaminated by food or drink. Four disposable swabs were used to scrape both buccal mucosae back and forth (at least 20 times) without touching the teeth to obtain buccal mucosal cells. The heads of the swabs were sealed in cryovials, immediately frozen in dry ice, transported to the laboratory and stored in a -80 °C freezer until needed. Genomic DNA was extracted from buccal mucosal cells with the TIANamp Genomic DNA Kit (Tiangen, Beijing, China) according to the manufacturer’s instructions.

The genotyping work was performed using a custom-designed 48-Plex single nucleotide polymorphism-scan kit (Genesky Biotechnologies, Inc., Shanghai, China). The kit was developed according to a patented genotyping technology by Genesky Biotechnologies, Inc., which was based on double ligation and multiplex fluorescence polymerase chain reaction (PCR). The genotyping processes were as follows: (1) a 1% agarose gel was used for quality inspection and to estimate the concentration. The concentration of the DNA sample ranged from 30 to 50 ng/µl. (2) Next, 2.5 µl of 4X DNA Lysis Buffer were added to 4 µl of DNA samples, the volume was increased to 10 µl with water, and samples were mixed and centrifuged. Then, the reaction was performed at 98 °C for 5 min and then immediately placed on ice. (3) Ten microliters of linking reaction premix were added to the frozen, degraded DNA sample, centrifuged for 30 s (3000 rpm), and immediately placed in the PCR instrument and cycled using the following program: 98℃ for 2 min, 5 cycles of 95 °C for 1 min, 58 °C for 3 h) and 94 °C for 2 min, followed by an incubation at 72 °C until subsequent reactions. The ligated products were then amplified by PCR using fluorescent primers using the following program: a hold cycle of 95 °C for 2 min, followed by 9 touchdown amplification cycles of 94 °C for 20 s, 62 °C (-0.5 °C/cycle) for 40 s, 72 °C for 1.5 min; 25 amplification cycles of 94 °C for 20 s, 57 °C for 40 s, and72°C for 1.5 min; and finally, an extension step at 68 °C for 1 h. (4) One microlitre of PCR product (after a 10-fold dilution) was mixed with 0.5 µl of Liz500 SIZE STANDARD and 8.5 µl of hi-di. After denaturation at 95 °C for 5 min, an ABI 3730XL sequencer was used to obtain the original data. (5) GeneMapper 4.1 (Applied Biosystems, USA) was used to analyse the data and record the fluorescence of markers and length of PCR products, as well as the corresponding single-nucleotide polymorphism (SNP) loci/allele information.

### Statistical analysis

Sex, ethnicity, parental education level, frequency of tooth brushing, frequency of sugary food intake, regular eating at school and genotype were the categorical variables and are presented as percentages. Categorical variables were compared between the two groups using Chi-square tests. Binary logistic regression analysis was performed to determine the associations between caries and the risk factors. Caries status was the dependent variable, whereby DMFT greater than zero is denoted as “1” and DMFT equal to zero as “0”. Chi-square tests and binary logistic regression analyses were performed using the Statistical Package for Social Sciences 25.0 software (SPSS, Chicago, IL, USA). Hardy–Weinberg equilibrium (HWE) was calculated using PLINK 1.90. A *P* value < 0.05 was considered statistically significant.

## Results

### Demographic characteristics and environmental factors

The kappa values for inter- and intra-examiner data during calibration were greater than 0.85. The demographic characteristics of the participants and environmental factors are shown in Table 1. A total of 1,061 children were enrolled in this study, including 586 caries-free children and 475 children with caries. The prevalence of caries was 44.8%. The proportion of female children with caries (51.0%) was higher than that of male children (39.3%) (*P* value < 0.001). Children who brushed their teeth more than once a day, ate sugary food more than once a week and did not regularly eat at school had higher rates of dental caries (*P* value < 0.05).

**Table 1:** Demographic characteristics and environmental factors of the study population stratified according to caries experience

Variable	Caries-free n (%)	Caries experience n (%)	*χ* ^*2*^	*P* value
**Total**	586 (55.2)	475 (44.8)		
**Sex**			14.755	< 0.001
Male	342 (60.7)	221 (39.3)		
Female	244 (49.0)	254 (51.0)		
**Ethnicity**			12.622	0.002
Han	135 (49.1)	140 (50.9)		
Zhuang	178 (52.0)	164 (48.0)		
Baikuyao	273 (61.5)	171 (38.5)		
**Parental education level**			3.638	0.056
Primary school and below	317 (58.1)	229 (41.9)		
Secondary school and above	269 (52.2)	246 (47.8)		
**Frequency of tooth brushing**			5.953	0.015
≤ once/day	363 (58.4)	259 (41.6)		
> once/day	223 (50.8)	216 (49.2)		
**Frequency of sugary food intake**			10.150	0.001
≤ once/week	217 (62.2)	132 (37.8)		
> once/week	369 (51.8)	343 (48.2)		
**Regularly eat at school**			7.025	0.008
Yes	345 (58.9)	241 (41.1)		
No	241 (50.7)	234 (49.3)		

### Analysis of risk factors

The variables (demographic characteristics and environmental factors) with *P* values < 0.05 were included in the binary logistic regression model (forward LR method) to determine the risk factors for caries. Female children had a 59.5% higher chance of having caries than male children (odds ratio [OR] = 1.595, 95% confidence interval (CI) = 1.246–2.042, *P* value < 0.001). Baikuyao children had a 35.7% lower chance of having caries than Han children (OR = 0.643, 95% CI = 0.471–0.879, *P* value = 0.006). Children who ate sugary foods more than once a week had a 34.0% higher probability of having caries than children who ate sugary foods at most once a week (OR = 1.340, 95% CI = 1.021–1.760, *P* value = 0.035) (Table 2).

**Table 2:** Binary logistic regression analysis of environmental risk factors with caries experience

Variable	OR	95% CI	*P* value
**Sex**			
Male	Ref		
Female	1.595	1.246–2.042	< 0.001
**Ethnicity**			
Han	Ref		
Zhuang	0.906	0.657–1.248	0.545
Baikuyao	0.643	0.471–0.879	0.006
**Frequency of sugary food intake**			
≤ once/week	Ref		
> once/week	1.340	1.021–1.760	0.035
**Constant**	0.198		< 0.001

Dependent variable: caries free (DMFT score = 0) and caries experience (DMFT score ≥ 1)

OR: odds ratio, CI: confidence interval

### Genetic analysis

All DNA samples were successfully genotyped. The minor allele frequencies (MAFs) of rs27565 and rs11362 were 0.472 and 0.280, respectively. The genotyped polymorphisms agreed with Hardy-Weinberg equilibrium (HWE) (Table 3).

**Table 3:** Descriptive statistics for the *PART1* rs27565 and *DEFB1* rs11362 genotypes

Chr	Gene	SNP	Allele	MAF	*P*_HWE_
5	*PART1*	rs27565	T/C	0.472	0.655
8	*DEFB1*	rs11362	C/T	0.280	0.737

Chr: chromosome, SNP: single-nucleotide polymorphism, MAF: minor allele frequency, HWE: Hardy-Weinberg equilibrium

Both the genotype and allele distributions of *PART1* rs27565 and *DEFB1* rs11362 were significantly different between caries-free children and children with caries experience (*P* value < 0.05) (Table 4).

**Table 4:** Genotype and allele distributions of the *PART1* rs27565 and *DEFB1* rs11362 polymorphisms between caries-free children and children with caries experience

SNP	Type	Variable	Caries-free	Caries experience	*χ* ^*2*^	*P* value
***PART1*** **rs27565**	Genotype				11.798	0.003
		TT	189 (32.3)	118 (24.8)		
		TC	282 (48.1)	227 (47.8)		
		CC	115 (19.6)	130 (27.4)		
	Allele				12.091	0.001
		T	660 (56.3)	463 (48.7)		
		C	512 (43.7)	487 (51.3)		
***DEFB1*** **rs11362**	Genotype				12.985	0.002
		CC	337 (57.5)	225 (47.4)		
		CT	208 (35.5)	196 (41.3)		
		TT	41 (7.0)	54 (11.4)		
	Allele				13.705	< 0.001
		C	882 (75.3)	646 (68.0)		
		T	290 (24.7)	304 (32.0)		

SNP: single-nucleotide polymorphism

The demographic and environmental risk factors identified above (sex, ethnicity and frequency of sugary food intake) were included as covariates in the binary logistic regression analyses (Enter method) to identify the association between genotypes and alleles of *PART1* rs27565 and *DEFB1* rs11362 with dental caries (dependent variable: DMFT score = 0 and DMFT score ≥ 1). The results are presented in Table 5. The codominant model showed that carriers of the *PART1* rs27565 CC genotype had an approximately 1.7-fold higher risk of caries than TT genotype carriers (OR = 1.692, 95% CI = 1.194–2.397, *P* value = 0.003), and carriers of the *DEFB1* rs11362 TT genotype also had a 1.7-fold higher risk of caries than CC genotype carriers (OR = 1.713, 95% CI = 1.084–2.708, *P* value = 0.021) (Table 5). Children who carried the C allele of the rs27565 polymorphism had a higher risk of caries in the dominant, additive and allelic models (OR = 1.338, 95% CI = 1.015–1.764, *P* value = 0.039; OR = 1.296, 95% CI = 1.089–1.543, *P* value = 0.003; and OR = 1.303, 95% CI = 1.094–1.911, *P* value = 0.003;, respectively). Children who carried the T allele of the rs11362 polymorphism also had a higher risk of caries in the dominant, additive and allelic models (OR = 1.364, 95% CI = 1.056–1.762, *P* value = 0.017; OR = 1.306, 95% CI = 1.072–1.590, *P* value = 0.008; and OR = 1.307, 95% CI = 1.074–1.592, *P* value = 0.008;, respectively).

**Table 5:** Association of the *PART1* rs27565 and *DEFB1* rs11362 genotypes with caries

SNP	Model	Variable	Caries-free n (%)	Caries experience n (%)		Univariate analysis			Multivariate analysis	
						**OR (95% CI)**	***P*** **value**		**OR (95% CI)**	P* **value**
***PART1*** **rs27565**	Codominant	TT	189 (32.3)	118 (24.8)		Ref			Ref	
		TC	282 (48.1)	227 (47.8)		1.289 (0.966–1.721)	0.085		1.198 (0.892–1.607)	0.230
		CC	115 (19.6)	130 (27.4)		1.811 (1.288–2.545)	0.001		1.692 (1.194–2.397)	0.003
	Dominant	TT	189 (32.3)	118 (24.8)		Ref			Ref	
		TC + CC	397 (67.7)	357 (75.2)		1.440 (1.099–1.888)	0.008		1.338 (1.015–1.764)	0.039
	Additive	–	–	–		1.343 (1.133–1.592)	0.001		1.296 (1.089–1.543)	0.003
	Allelic	T	660 (56.3)	463 (48.7)		Ref			Ref	
		C	512 (43.7)	487 (51.3)		1.356 (1.142–1.610)	0.001		1.303 (1.094–1.911)	0.003
***DEFB1*** **rs11362**	Codominant	CC	337 (57.5)	225 (47.4)		Ref			Ref	
		CT	208 (35.5)	196 (41.3)		1.411 (1.090–1.827)	0.009		1.300 (0.995–1.700)	0.055
		TT	41 (7.0)	54 (11.4)		1.973 (1.271–3.062)	0.002		1.713 (1.084–2.708)	0.021
	Dominant	CC	337 (57.5)	225 (47.4)		Ref			Ref	
		CT + TT	249 (42.5)	250 (52.6)		1.504 (1.179–1.918)	0.001		1.364 (1.056–1.762)	0.017
	Additive	–	–	–		1.407 (1.167–1.696)	< 0.001		1.306 (1.072–1.590)	0.008
	Allelic	C	882 (75.3)	646 (68.0)		Ref			Ref	
		T	290 (24.7)	304 (32.0)		1.431 (1.183–1.731)	< 0.001		1.307 (1.074–1.592)	0.008

SNP: single-nucleotide polymorphism, OR: odds ratio, CI: confidence interval, *: Adjusted for sex, ethnicity and frequency of sugary food intake

The frequency of sugary food intake was a significant environmental risk factor for caries in the present study. A binary logistic regression model (Enter method) was also used to analyse the effects of different combinations of genetic polymorphisms (*PART1* rs27565 and *DEFB1* rs11362) and the frequency of sugary food intake on caries. After adjusting for sex and ethnicity, carriers of the *PART1* rs27565 TC or CC genotype who ate sugary food more than once a week had a 1.6-fold increase in their risk of dental caries compared to TT carriers who ate sugary food at most once a week (OR = 1.579, 95% CI = 1.032–2.414, *P* value = 0.035). Carriers of the *DEFB1* rs11362 CT or TT genotype who ate sugary food more than once a week had a 2.1-fold increased risk of caries than CC carriers who ate sugary food at most once a week (OR = 2.057, 95% CI = 1.438–2.940, *P* value < 0.001) (Table 6).

**Table 6:** Effects of different combinations of genetic polymorphisms (*PART1* rs27565 and *DEFB1* rs11362) and the frequency of sugary food intake on caries

SNP	Combination	Caries-free n (%)	Caries experience n (%)	OR	95% CI	P* value
***PART1*** **rs27565**						
	TT/sugary food intake ≤ once/week	77 (13.1)	43 (9.1)	Ref		
	TT/sugary food intake > once/week	112 (19.1)	75 (15.8)	1.054	0.649–1.711	0.832
	TC + CC/sugary food intake ≤ once/week	140 (23.9)	89 (18.7)	1.084	0.682–1.723	0.734
	TC + CC/sugary food intake > once/week	257 (43.9)	268 (56.4)	1.579	1.032–2.414	0.035
***DEFB1*** **rs11362**						
	CC/sugary food intake ≤ once/week	132 (22.5)	72 (15.2)	Ref		
	CC/sugary food intake > once/week	205 (35.0)	153 (32.2)	1.324	0.926–1.894	0.124
	CT + TT/sugary food intake ≤ once/week	85 (14.5)	60 (12.6)	1.297	0.835–2.015	0.247
	CT + TT/sugary food intake > once/week	164 (28.0)	190 (40.0)	2.057	1.438–2.940	< 0.001

SNP: single-nucleotide polymorphism, OR: odds ratio, CI: confidence interval

*: Adjusted for sex and ethnicity

## Discussion

This study identified the relationship of *PART1* and *DEFB1* polymorphisms with caries prevalence among 12-year-old children in Nandan County, Guangxi, China. Our results showed that carriers of the *PART1* rs27565 C allele and the *DEFB1* rs11362 T allele had higher risks of dental caries.

In this study, Baikuyao children had a lower caries prevalence than Han and Zhuang children, potentially due to their lower frequency of sugary food intake. Sugary food intake is an important risk factor for dental caries [2]. Our previous study reported lower parental education levels for Baikuyao children [31]. A lower parental education level often indicates a lower household income [32]. As most of the Baikuyao population could barely afford their household expenses, they might not have much money to buy sugary food for their children [31]. According to the published literature, the relationship between income and added sugar intake is curvilinear, increasing from very low-income to middle-income households, followed by a decrease among children from high-income households [33]. Moreover, most Baikuyao children often eat regularly at school, which may reduce their access to sugar and lead to their lower frequency of sugary food intake [33]. Nevertheless, other factors may be involved in different caries statuses among this population, and we will expand the sample in future studies.

The multivariate analyses indicated that the *PART1* rs27565 polymorphism may be a risk factor for caries. *PART1* encodes a long noncoding RNA (lncRNA). Transcription factors with immune function, such as nuclear factor interleukin-3 (NFIL3)-regulated, nuclear factor of activated T cells1 (NFATC1) and regulatory factor X1 (RFX1), may bind to *PART1* and then be coexpressed, resulting in antibacterial and immune activity [12]. The expression of *PART1* in the oral cavity affects the proliferation of oral squamous cell carcinoma [34] and was also speculated to affect dental caries by regulating immunity and oral microbes [12]. We speculated that compared with the rs27565 T allele, the C allele is associated with a lower ability of immune-related transcription factors to bind *PART1* and a lower level of coexpressed products. Therefore, individuals with the rs27565 C allele may be more sensitive to plaque accumulation, resulting in an increased risk of caries. Our results were consistent with those reported by Shimizu, who found that carriers of the *PART1* rs27565 C allele had a higher risk of caries in the Filipino family [12]. The study by Kelly also revealed that the C allele frequency was higher in the caries group [14]. However, studies explaining the biological mechanism underlying the association between the *PART1* rs27565 polymorphism and caries occurrence are insufficient.

The *DEFB1* rs11362 T allele was also a risk factor for caries in the current study. *DEFB1* rs11362 is a site in the promoter. SNPs in the promoter region may alter transcriptional activity compared with that region in the wild type gene [35]. Therefore, we speculate that the *DEFB1* rs11362 T allele may reduce the expression level of β-defensin 1, resulting in lower antibacterial ability and thereby increasing the risk of caries. The studies by Ozturk, Yildiz and Wu supported this hypothesis. They found that carriers of the *DEFB1* rs11362 TT genotype had a higher risk of caries [19, 21, 22]. A meta-analysis published in 2020 also showed that individuals with the TT genotype had a seven times higher risk of caries in permanent dentition than individuals with the CC genotype [11]. However, our results are inconsistent with those reported by Krasone and Navarra, who found that CC genotype carriers had a higher risk of caries [23, 24]. Possible explanations for these inconsistencies mainly included differences in genetic heterogeneity. Our results suggested that the *DEFB1* rs11362 polymorphism may be a potential biomarker for caries among 12-year-old children in Nandan County, Guangxi, China.

We also found that carriers of the *PART1* rs27565 TC + CC genotype and *DEFB1* rs11362 CT + TT genotype who ate sweets more than once a week had a higher risk of caries, with ORs of 1.579 and 2.057, respectively. Although this result does not prove the interaction between genes and environmental factors, it suggests a stronger risk effect on caries when the two risk factors are present at the same time.

Our results supported the possibility of *DEFB1* rs11362 and *PART1* rs27565 polymorphisms as caries risk markers and provided a basis for identifying caries-susceptible populations and developing caries prevention strategies. However, our study has several limitations. First, we did not perform a subgroup analysis stratified by ethnicity because of the small sample size. Second, all independent variables were categorical data. Compared with continuous variable, categorical variables may reduce some available information, and different cut-off points of categorizing continuous data may lead to slightly different results. Third, environmental factors are very complex. However, insufficient environmental factors were included in this study, and thus more of them should be included in future studies.

## Conclusion

*PART1* rs27565 and *DEFB1* rs11362 polymorphisms might be associated with caries in 12-year-old children in Nandan County, Guangxi, China. Carriers of the *PART1* rs27565 TC + CC genotype and the *DEFB1* rs11362 CT + TT genotype who ate sugary food more than once a week had a higher risk of developing dental caries. *PART1* and *DEFB1* polymorphisms may thus be potential risk factors for a childhood caries diagnosis. These results must be confirmed in other populations with a larger sample size.

## Data Availability

The data and analysis for the current study are available from the corresponding author on reasonable request.

## Abbreviations

DMFT: Decayed, Missing and Filled Teeth
Chr: chromosome SNP:single-nucleotide polymorphism
OR: odds ratio
CI: confidence interval
MAF: minor allele frequency
HWE: Hardy-Weinberg equilibrium
